# Robust Tracking Control of the Euler–Lagrange System Based on Barrier Lyapunov Function and Self-Structuring Neural Networks

**DOI:** 10.1155/2021/1277349

**Published:** 2021-10-12

**Authors:** Yi Wang, He Ma, Weidong Wu

**Affiliations:** ^1^College of Medicine and Biological Information Engineering, Northeastern University, Shenyang 110016, China; ^2^State Key Laboratory of Material Processing and Die and Mould Technology and School of Materials Science and Engineering, Huazhong University of Science and Technology, Wuhan 430074, China

## Abstract

This article studies the robust tracking control problems of Euler–Lagrange (EL) systems with uncertainties. To enhance the robustness of the control systems, an asymmetric tan-type barrier Lyapunov function (ATBLF) is used to dynamic constraint position tracking errors. To deal with the problems of the system uncertainties, the self-structuring neural network (SSNN) is developed to estimate the unknown dynamics model and avoid the calculation burden. The robust compensator is designed to estimate and compensate neural network (NN) approximation errors and unknown disturbances. In addition, a relative threshold event-triggered strategy is introduced, which greatly saves communication resources. Under the proposed robust control scheme, tracking behavior can be implemented with disturbance and unknown dynamics of the EL systems. All signals in the closed-loop system are proved to be bounded by stability analysis, and the tracking error can converge to the neighborhood near the origin. The numerical simulation results show the effectiveness and the validity of the proposed robust control scheme.

## 1. Introduction

Many practical systems can be represented by the El system, such as robotic manipulator [1], hydraulic system [2], and underwater marine system [3]. Therefore, due to its wide application, nonlinear Euler–Lagrange systems are a significant class of nonlinear systems. However, because of the unknown disturbances, the model uncertainties and the actuator communication limit always exist, and some traditional control methods are difficult to obtain satisfactory control performance. Therefore, innovation and development with high precision and high applicability control methods are urgent.

The research on robust control of the El system has always been a hot topic [4–7]. Generally, when a system works under uncertain disturbances, we need to improve the robustness of the control as much as possible. Some scholars have studied the trajectory tracking method of EL systems; the common methods include the backstepping technique [8], dynamics surface control (DSC) [9], robust control [10, 11], adaptive control [12, 13], sliding mode control [14], and learning control [15]. Among them, the error restriction method can effectively enhance the robustness of the control. In addition, considering the control security issues cannot be ignored, generally in the form of output constraints. Violation of these constraints not only leads to performance degradation but also causes system corruption. In most studies, BLF is an effective solution for the constraint problem [16–18]. In [19], the guaranteed performance control problem for EL systems with actuator faults is investigated, and the BLF is introduced to handle the performance constraints problems. In [20, 21], a log-type BLF is employed to ensure that the full-state constraints for an EL system with uncertain dynamics. In [22], the BLF-based control method is proposed for robotic systems with full-state constraints, which demonstrated that the BLF design method has advantages in dealing with state constraint problems of the El system. For research position constraint problems, a BLF-based controller is proposed for the marine vessel with uncertainty in [23], which also demonstrates the superiority of BLF in the El system design. The BLF technique can dynamically constrain the error within the specified range and guaranteed the performance of tracking control, which enhances the robustness of the control.

In practice, the influence of unknown disturbances on control is a serious problem. Some identification methods have been proposed to estimate the effects of uncertainties, such as the adaptive observers and compensate methods. The disturbance observer [24, 25] is a useful tool for nonlinear systems to identify unknown external disturbances, and some works have been applied to solve the problem of El system resisting disturbances [26, 27]. However, EL system model parameters are typically dynamically changed; they are difficult to obtain dynamics parameters accurately. The uncertainty seriously affects the stability and control accuracy of the EL system; therefore, the problem of identification of uncertain models is needed to be studied urgently. Some learning estimation methods have been proposed to approximate the system uncertainty, such as neural networks, fuzzy logic, and machine learning. The NN is often used to estimate unknown nonlinear dynamics models because of its good approximation ability. In [28–30], the adaptive method is combined with NN to design control strategies for a class of uncertain nonlinear systems. In [31, 32], the adaptive NN is used to estimate the uncertainty of El system in tracking or cooperative control. An adaptive multilayer NN is developed to estimate the uncertainty and a novel saturated prescribed performance controller for EL dynamic systems in [33]. In [34], to reduce the calculation burden, the adaptive NNs with the epsilon-modification updating laws are developed to approximate the compounded uncertain vector for EL systems. In [35], a self-structuring NN is designed to estimate the uncertain dynamics of each node of multiagents. Because NN has good learning performance, it has become the main tool to estimate system uncertainty.

Furthermore, in most cases, the bandwidth of the actuator communication network is limited. In order to use available resources reasonably, it is very important to design save resource controllers. It is worth noting that the event-triggered strategy is an effective way to reduce the actuator resources. In the event-triggered strategy, the control signal is updated only on some discrete trigger time to implement the aperiodic signal update. The trigger time is calculated based on some condition of the system state, which is also known as a trigger condition. This strategy makes the system have no complete transmission state throughout the time period and reduces the calculation workload and the use of communication channels [36, 37]. In [38], an adaptive control method is utilized to solve the unknown system parameters, and the new triggering mechanism is proposed to increase the executive efficiency of the controller. An event-triggered observer was designed for the estimation of the system states, and the dynamic event-triggered sliding mode controller is designed for a class nonlinear dynamic systems [39]. The sliding mode control method combines with event-triggered strategy, and a robust trajectory tracking controller is designed for uncertain EL systems [40]. In [41], a fully distributed event-triggered finite-time consensus controller is designed for EL systems, which can enable each agent to complete consistency tracking after a settling time.

Inspired by the above researches, the purpose of this paper is to design a robust track control strategy for EL systems with uncertainties. The ATBLF method is employed to constraint tracking errors, which can enhance the robustness of the tracking control. The adaptive NN is used to estimate the uncertainties of EL systems, and the self-structure mechanism is designed to reduce the calculation burden. The compensator is designed to estimate NN approximate error and the disturbances, which can improve the tracking accuracy. An event-triggered strategy is adopted to save actuator resources. The main contributions are summarized as follows:

1. To ensure the robustness of the tracking control, the control strategy design is divided into two layers. The ATBLF method is introduced to construct virtual control law at the kinematic level, it makes the position error guaranteed in a certain boundary, and the robustness of tracking is enhanced. In terms of kinetics, the adaptive NN is employed to estimate the uncertainty of EL systems. The NN approximate errors and unknown disturbances can compensate by a designed compensator, which ensures tracking stability
2. In order to improve the practicability of the control systems, a self-structure mechanism is developed to adjust NN approximation performance, which can appropriately find optimal NN structures and avoid excessive calculation burden. In addition, an event-triggered strategy is adopted to reduce the communication bandwidth and effectively save communication resources

This paper is organized as follows. The problem formulation of EL systems is introduced in Section 2. The main results of the design of SSNN and the robust tracking control strategy on the EL systems are in Section 3.  Section 4 presents numerical simulation results. The conclusions of this paper are presented in Section 5.

Notation: *λ*_max_(*·*) and *λ*_min_(*·*) denote the largest and smallest eigenvalue, respectively. *ℝ*^*n*^ and *ℝ*^*n*×*n*^ denote *n* dimensional column vectors and the *n* × *n* real matrices, respectively. ‖*·*‖_*F*_ and ‖*·*‖ represent the Frobenius norm and the Euclidean norm. diag{*·*} represents a block-diagonal matrix.

## 2. Problem Formulation

### 2.1. System Model

Consider the uncertain EL systems with external disturbances, which is

(1) $$\begin{array}{c} M\left(q\right)\ddot{q}+C\left(q,\dot{q}\right)\dot{q}+G\left(q\right)=\tau \left(t\right)-d\left(t\right), \end{array}$$

where *q*, $\dot{q}$, and $\ddot{q}\in {\mathbb{R}}^{n}$ denote the position, velocity, and acceleration vectors, respectively; *G*(*q*) ∈ *ℝ*^*n*^ denotes the gravitational force, $C\left(q,\dot{q}\right)\in {\mathbb{R}}^{n}$ denotes the Coriolis and centripetal torques, *M*(*q*) ∈ *ℝ*^*n*×*n*^ denotes a symmetric inertia matrix, *d*(*t*) ∈ *ℝ*^*n*^ is the external disturbances of the systems caused by the environment and human beings, and *τ* ∈ *ℝ*^*n*^ is the control input.

**Property 1 (see [42]).**

*M*(*q*), $C\left(q,\dot{q}\right)$, and *G*(*q*) in the dynamic system are all bound, and the matrix $\dot{M}\left(q\right)-2C\left(q,\dot{q}\right)$ is skew-symmetric; i.e., $\varsigma^{T}\left[\dot{M}\left(q\right)-2C\left(q,\dot{q}\right)\right]\varsigma =0$ for any *ς* ∈ *ℝ*^*p*^.

### 2.2. Control Objective

The reference trajectory is defined as *q*_*d*_=[*q*_*d*1_,…,*q*_*dn*_]^*T*^, which is time-varying twice-differentiable, and the tracking errors are defined as *e*_*p*_=*q* − *q*_*d*_. The goal is to design a robust tracking controller for the EL systems to track the reference trajectory and to keep the tracking error *e*_*p*_ constraints within a time-varying asymmetric bounded range as follows:

(2) $$\begin{array}{c} -L_{L}\left(t\right)<e_{p}\left(t\right)<L_{U}\left(t\right),\;\forall t>0, \end{array}$$

where *L*_*L*_ ∈ *ℝ*^*n*^ and *L*_*U*_ ∈ *ℝ*^*n*^ denote the constraint bounded functions on the tracking error *e*_*p*_, and the initial condition satisfies −*L*_*L*_(0) < *e*_*p*_(0) < *L*_*U*_(0).

**Assumption 1 .**

The disturbance *d*_*i*_ and its first derivative ${\dot{d}}_{i}$ are bounded, such that ${\left\|d_{i}\right\|}_{\infty }\leq {\bar{d}}_{i}$ and ${\left\|{\dot{d}}_{i}\right\|}_{\infty }\leq {\bar{d}}_{id}$, where ${\bar{d}}_{i}$ and ${\bar{d}}_{id}$ are unknown positive constants.

**Remark 1 .**

Disturbance may occur in the form of variable friction or load, which is often variable and unpredictable, and the energy is limited. If it is infinite energy, it will destroy the control system. Therefore, Assumption 1 is reasonable.

## 3. Main Results

In this section, the design process of the robust tracking control strategy for EL systems based on BLF and SSNN is introduced. The SSNN is developed to estimate unknown model dynamics. The TABLF is applied to deal with error time-varying constraint problems. The compensator is designed to estimate unknown disturbances and NN estimate errors. An event-triggered strategy is adopted to reduce actuator communication pressure.

### 3.1. Self-Structuring Neural Networks

**Figure 1:**
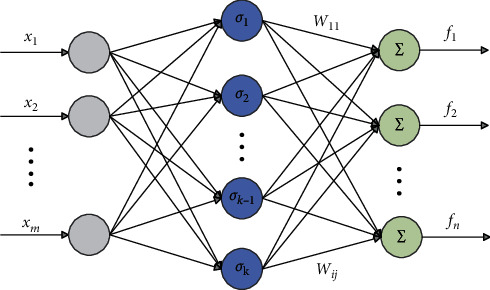
Structure of the NN.

In this article, the radial basis function (RBF) NN is applied to approximate unknown nonlinear dynamics. The RBFNN is composed of the output layer, hidden layer, and input layer, and its structure is shown in Figure 1.

The RBFNN output is expressed as

(3) $$\begin{array}{c} f\left(\chi \right)=W^{*T}\sigma^{*}\left(\chi \right)+\varepsilon , \end{array}$$

where *σ*^*∗*^(*χ*)=[*σ*_1_^*∗*^(*χ*), *σ*_2_^*∗*^(*χ*),…,*σ*_*k*_^*∗*^(*χ*)]^*T*^ is the activation function vector, *χ*=[*χ*_1_, *χ*_2_,…,*χ*_*m*_]^*T*^ ∈ *ℝ*^*m*^ is the input vector, there are *k* neurons here, and *W*^*∗*^=[*W*_1_^*∗*^, *W*_2_^*∗*^, ⋯,*W*_*k*_^*∗*^]^*T*^ is the ideal NN weight vector. *ε* represents the NN approximation error, where the activation function is selected as the Gaussian function:

(4) $$\begin{array}{c} \sigma_{j}^{*}\left(\chi \right)=\exp\left[-\frac{{\left\|\chi -\mu_{j}\right\|}^{2}}{h_{j}{}^{2}}\right],\;j=1,\ldots ,k, \end{array}$$

where *μ*_*j*_ and *h*_*j*_ represent the center and width of the Gaussian function, respectively.

About RBFNN, the more the neuron nodes are selected, the more accurate the approximation. However, more neurons mean that the system has a more computational burden, and some neurons are invalid when the nonlinear function is not complex. Therefore, we design a self-structuring mechanism for NN to change the approximation structure, which can determine whether to split neurons or eliminate neurons depending on the complexity of the actual nonlinear function. The aim is to split more effectively activation neurons and delete less activated neurons to obtain good approximation performance of NN.

The optimization method of NN is proposed. Define a splitting threshold *S*_*s*_ ∈ (0,1) and eliminate threshold *S*_*e*_ ∈ (0,1), where *S*_*s*_ > *S*_*e*_.

The splitting strategy is to judge whether the neuron with the highest activation function is more than the threshold, which defines the maximum degree $\sigma_{M}=\underset{1\leq j\leq k}{\max}\sigma_{j}$; if *σ*_*M*_ ≤ *S*_*s*_, that means the activity does not reach the ideal value; then, the new neurons need to be split. The newly splitting neuron is defined as *j*′; the parameter of the new neuron is

(5) $$\begin{array}{c} \left\{\begin{array}{c} \mu_{j\prime }=\frac{\chi +\mu_{j}}{2},\\ h_{j\prime }=h_{j},\\ W_{j\prime }=0. \end{array}\right. \end{array}$$

The neuron decay parameter is defined as *I*_*j*_; it follows the rules

(6) $$\begin{array}{c} I_{j}=\left\{\begin{array}{cc} \psi I_{j}, & \text{if }\sigma_{j}\leq \sigma_{e},\\ 1, & \text{if }\sigma_{j}>\sigma_{e}, \end{array}\right.\;j=1,\ldots ,k, \end{array}$$

where *ψ* is a proportion parameter and *σ*_*e*_ denotes the inactive bounded function. The elimination strategy is proposed. When the activation function *σ*_*j*_ is less than a threshold *σ*_*e*_, the neuron decay parameter *I*_*j*_ will decrease. When *I*_*j*_ ≤ *S*_*e*_, the *j*_*th*_ neuron is pruned.

**Figure 2:**
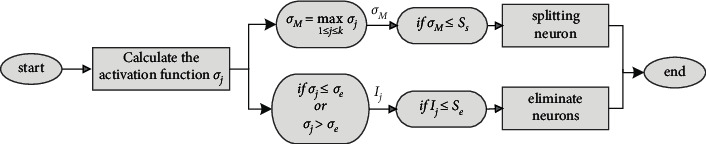
Self-structuring algorithm flowchart.

The logic block diagram of self-structuring strategy is shown in Figure 2.

**Assumption 2 (see [43]).**

The ideal NN weight is bounded such that ‖*W*‖_*F*_ ≤ *W*_*m*_, where *W*_*m*_ are unknown positive constants.

**Remark 2 .**

Some existing works [29, 30] show that the more the number of neurons, the better the approximation effect of NN. It is worth noting that not all neurons are effective neurons, which will bring more calculation burden to the control system. Therefore, a self-structuring mechanism with a flexible structure is proposed in this paper. The advantages of SSNN including the structuring of NN can be adjusted online without new membership functions and rules, and the computation can be effectively reduced.

### 3.2. Controller Design

The design process is divided into two steps.

**Step 1 .**

The asymmetrical errors virtual controller is designed.

Define the tracking error vector *z*_1_ ∈ *ℝ*^*n*^ and *z*_2_ ∈ *ℝ*^*n*^ as

(7) $$\begin{array}{c} \left\{\begin{array}{c} z_{1}=e_{p},\\ z_{2}=\dot{q}-v_{d}, \end{array}\right. \end{array}$$

where *z*_1_=[*z*_11_,…,*z*_1*n*_]^*T*^, *z*_2_=[*z*_21_,…,*z*_2*n*_]^*T*^, and *v*_*d*_ ∈ *ℝ*^*n*^ is a filtered control signal to be specified later.

Taking the time derivation of tracking error combined with EL system (1) yields

(8) $$\begin{array}{c} \left\{\begin{array}{c} {\dot{z}}_{1}=\dot{q}-{\dot{q}}_{d},\\ M{\dot{z}}_{2}=\tau -d-C\dot{q}-G-M{\dot{v}}_{d}, \end{array}\right. \end{array}$$

and the time-varying error constraint problem can be solved by the BLF method. Consider the asymmetric tan-type BLF (ATBLF) as follows [44]:

(9) $$\begin{array}{c} V_{1}=\sum_{i=1}^{n}\left(1-q\left(z_{1i}\right)\right)\frac{L_{Li}{}^{2}}{\pi }\tan\left(\frac{\pi z_{1i}{}^{2}}{2L_{Li}{}^{2}}\right)+q\left(z_{1i}\right)\frac{L_{Ui}{}^{2}}{\pi }\tan\left(\frac{\pi z_{1i}{}^{2}}{2L_{Ui}{}^{2}}\right), \end{array}$$

(10) $$\begin{array}{c} q\left(z_{1i}\right)=\left\{\begin{array}{cc} 1, & z_{1i}>0,\\ 0, & z_{1i}\leq 0, \end{array}\right.\;i=1,\ldots ,n. \end{array}$$

Computing the time derivative of *V*_1_ yields

(11) $$\begin{array}{c} {\dot{V}}_{1}=\sum_{i=1}^{n}\left(1-q\left(z_{1i}\right)\right)\left[\frac{2L_{Li}{\dot{L}}_{Li}}{\pi }\tan\left(\frac{\pi z_{1i}{}^{2}}{2L_{Li}{}^{2}}\right)+\Lambda_{Li}{\dot{z}}_{1i}-\Lambda_{Li}z_{1i}\frac{{\dot{L}}_{Li}}{L_{Li}}\right]+q\left(z_{1i}\right)\left[\frac{2L_{Ui}{\dot{L}}_{Ui}}{\pi }\tan\left(\frac{\pi z_{1i}{}^{2}}{2L_{Ui}{}^{2}}\right)+\Lambda_{Ui}{\dot{z}}_{1i}-\Lambda_{Ui}z_{1i}\frac{{\dot{L}}_{Ui}}{L_{Ui}}\right], \end{array}$$

where Λ_*Li*_=*z*_1*i*_/cos^2^(*πz*_1*i*_^2^/2*L*_*Li*_^2^), Λ_*Ui*_=*z*_1*i*_/cos^2^(*πz*_1*i*_^2^/2*L*_*Ui*_^2^), the initial state satisfies −*L*_*Li*_(0) < *z*_1*i*_(0) < *L*_*Ui*_(0), and *L*_*Li*_ and *L*_*Ui*_ are the presetting boundaries. Define Ξ_Λ*i*_=(1 − *q*(*z*_1*i*_))Λ_*Li*_+*q*(*z*_1*i*_)Λ_*Ui*_.

**Remark 3 .**

For the formation of asymmetric tan-type BLF, which is shown in (9), we have

(12) $$\begin{array}{c} \left\{\begin{array}{c} \underset{z_{1i}⟶0^{+}}{\lim}V_{1}=\underset{z_{1i}⟶0^{-}}{\lim}V_{1}=0,\\ \underset{z_{1i}⟶L_{Li}}{\lim}V_{1}=\underset{z_{1i}⟶L_{Ui}}{\lim}V_{1}=\infty , \end{array}\right. \end{array}$$

where *V*_1_ is differentiable and continuous and the state *z*_1*i*_ follows −*L*_*Li*_(*t*) < *z*_1*i*_(*t*) < *L*_*Ui*_(*t*). When there are system states without constraints, such as *L*_*Li*_⟶*∞* and *L*_*Ui*_⟶*∞*, using L'Hospital theory:

(13) $$\begin{array}{c} \underset{L_{Li}⟶\infty ,L_{Ui}⟶\infty }{\lim}V_{1}=\frac{1}{2}\sum_{i=1}^{n}z_{1i}^{2}. \end{array}$$

Then, we proposed the constraint virtual controller as

(14) $$\begin{array}{c} \alpha_{i}=\frac{-\left(k_{1i}+2\Xi_{Li}\right)\left[\left(1-q\left(z_{1i}\right)\right)L_{Li}^{2}\sin\left(\pi z_{1i}^{2}/L_{Li}^{2}\right)+q\left(z_{1i}\right)L_{Ui}^{2}\sin\left(\pi z_{1i}^{2}/L_{Ui}^{2}\right)\right]}{2\pi z_{1i}}+\Xi_{Li}z_{1i}+{\dot{q}}_{di},\;i=1,\ldots ,n, \end{array}$$

where *k*_1*i*_ > 0 is a positive design constant, $\Xi_{Li}=\left(1-q\left(z_{1i}\right)\right)\left({\dot{L}}_{Li}/L_{Li}\right)+q\left(z_{1i}\right)\left({\dot{L}}_{Ui}/L_{Ui}\right)$, and *k*_1_=diag{*k*_11_,…, *k*_1*n*_} is a positive gain matrix.

In order to avoid the differential explosion of virtual control law, the DSC method is introduced. The filtered control signal *v*_*d*_ is as follows:

(15) $$\begin{array}{c} t_{d}{\dot{v}}_{d}+v_{d}=\alpha_{d},v_{d}\left(0\right)=\alpha_{d}\left(0\right), \end{array}$$

where *α*_*d*_=*α*+*t*_*d*_Ξ_Λ_, and *t*_*d*_ is a time constant. Define the filtering error *e*_*f*_=*v*_*d*_ − *α* ∈ *ℝ*^*n*^, and take derivatives of *e*_*f*_:

(16) $$\begin{array}{c} {\dot{e}}_{f}=-\frac{e_{f}}{t_{d}}-\Xi_{\Lambda }-N\left(\cdot \right), \end{array}$$

where $\dot{\alpha }≜N\left(\cdot \right)$ with $N\left(q,\dot{q},L_{L},{\dot{L}}_{L},L_{U},{\dot{L}}_{U},z_{1},z_{2},e_{f}\right)\in {\mathbb{R}}^{n}$ being an unknown continuous function which has a maximum value $\bar{N}\in {\mathbb{R}}^{n}$.

Substituting (14) and (15) into (11), the following can be obtained:

(17) $$\begin{array}{c} {\dot{V}}_{1}\leq \sum_{i=1}^{n}-\left(k_{1i}\right)\left[\left(1-q\left(z_{1i}\right)\right)\frac{L_{Li}^{2}}{\pi }\tan\left(\frac{\pi z_{1i}^{2}}{2L_{Li}{}^{2}}\right)+q\left(z_{1i}\right)\frac{L_{Ui}^{2}}{\pi }\tan\left(\frac{\pi z_{1i}^{2}}{2L_{Ui}^{2}}\right)\right]+\Xi_{\Lambda i}\left(z_{2i}+e_{fi}\right). \end{array}$$

Consider the following Lyapunov function:

(18) $$\begin{array}{c} V_{2}=V_{1}+\frac{1}{2}e_{f}^{T}e_{f}. \end{array}$$

And, take the derivative of *V*_2_ and combine with (15) and (16) to obtain

(19) $$\begin{array}{c} {\dot{V}}_{2}\leq \sum_{i=1}^{n}-\left(k_{1i}\right)\left[\left(1-q\left(z_{1i}\right)\right)\frac{L_{Li}^{2}}{\pi }\tan\left(\frac{\pi z_{1i}^{2}}{2L_{Li}^{2}}\right)+q\left(z_{1i}\right)\frac{L_{Ui}^{2}}{\pi }\tan\left(\frac{\pi z_{1i}^{2}}{2L_{Ui}^{2}}\right)\right]+\Xi_{\Lambda i}z_{2i}-\frac{e_{fi}^{2}}{t_{d}}-e_{fi}N_{i}\left(\cdot \right). \end{array}$$

**Step 2 .**

The robust controller based on SSNN and event triggers is designed.

Consider the following Lyapunov function:

(20) $$\begin{array}{c} V_{3}=V_{2}+\frac{1}{2}z_{2}^{T}Mz_{2}. \end{array}$$

According to (7) and (8), EL system (1) can be written as

(21) $$\begin{array}{c} M\left(q\right){\dot{z}}_{2}+C\left(q,\dot{q}\right)z_{2}=\tau -M\left(q\right){\dot{v}}_{d}-C\left(q,\dot{q}\right)v_{d}-G\left(q\right)-d. \end{array}$$

Taking the derivative of *V*_3_, we can obtain

(22) $$\begin{array}{c} {\dot{V}}_{3}={\dot{V}}_{2}+\frac{1}{2}z_{2}^{T}\dot{M}z_{2}+z_{2}^{T}M{\dot{z}}_{2}. \end{array}$$

Combined with Property 1, one has $z_{2}^{T}\left[\dot{M}\left(q\right)-2C\left(q,\dot{q}\right)\right]z_{2}=0$. Moreover, substituting (21) into (22) yields

(23) $$\begin{array}{c} {\dot{V}}_{3}\leq \sum_{i=1}^{n}-\left(k_{1i}\right)\left[\left(1-q\left(z_{1i}\right)\right)\frac{L_{Li}^{2}}{\pi }\tan\left(\frac{\pi z_{1i}^{2}}{2L_{Li}^{2}}\right)+q\left(z_{1i}\right)\frac{L_{Ui}^{2}}{\pi }\tan\left(\frac{\pi z_{1i}^{2}}{2L_{Ui}^{2}}\right)\right]+\Xi_{\Lambda i}z_{2i}-\frac{e_{fi}^{2}}{t_{d}}-e_{fi}N_{i}\left(\cdot \right)+z_{2}^{T}\left[\tau -M\left(q\right)\left(-\frac{e_{f}}{t_{d}}-\Xi_{\Lambda }\right)-C\left(q,\dot{q}\right)v_{d}-G\left(q\right)-d\right]. \end{array}$$

Define function $f=M\left(q\right)\left(-e_{f}/t_{d}-\Xi_{\Lambda }\right)+C\left(q,\dot{q}\right)v_{d}+G\left(q\right)\in {\mathbb{R}}^{n}$ . However, the parameters *G*, $C\left(q,\dot{q}\right)$, and *M* are hard to obtain in the practice scene. Hence, the NN is employed to handle the uncertainty model as follows:

(24) $$\begin{array}{c} f=W^{T}\sigma \left(\chi \right)+\varepsilon , \end{array}$$

where the input of NN is selected as $\chi ={\left[q,\dot{q},v_{d},{\dot{v}}_{d}\right]}^{T}$, *W*=[*W*_1_,…,*W*_*i*_]^*T*^ is the NN weight matrix, and *ε* denotes the estimated error, which is bounded and satisfied, $\left|\varepsilon \right|\leq \bar{\varepsilon }$, where $\bar{\varepsilon }={\left[{\bar{\varepsilon }}_{1},\ldots ,{\bar{\varepsilon }}_{n}\right]}^{T}$ is an unknown positive constant vector. In addition, define the unknown parameters vector *δ*=[*δ*_1_,…,*δ*_*n*_]^*T*^, where $\delta_{i}={\bar{\varepsilon }}_{i}+{\bar{d}}_{i}$ is the unnecessary systems error. Then, a compensator is designed as follows:

(25) $$\begin{array}{c} c_{\text{aux},i}=-\frac{{\hat{\delta }}_{i}}{2w_{i}}z_{2i},\;i=1,\ldots ,n. \end{array}$$

The robust control laws are designed as follows:

(26) $$\begin{array}{c} \varpi_{i}\left(t\right)=-\left(1+\Delta_{i}\right)\left(\alpha_{vi}\tanh\left(\frac{z_{2i}\alpha_{vi}}{\kappa_{i}}\right)+{\bar{m}}_{i}\tanh\left(\frac{z_{2i}{\bar{m}}_{i}}{\kappa_{i}}\right)\right), \end{array}$$

(27) $$\begin{array}{c} \alpha_{v}=-k_{2}z_{2}-\Xi_{\Lambda }+{\hat{W}}^{T}\sigma \left(\chi \right)+c_{\text{aux}}, \end{array}$$

where *k*_2_=diag{*k*_21_,…, *k*_2*n*_}, Ξ_Λ_=[Ξ_Λ1_,…,Ξ_Λ*n*_]^*T*^, $\hat{W}={\left[{\hat{W}}_{1},\ldots ,{\hat{W}}_{n}\right]}^{T}$ is the NN weight matrix estimate value, and *c*_aux_=[*c*_aux,1_,…,*c*_aux,*n*_]^*T*^ .

The updated law $\hat{W}$ and $\hat{\delta }$ are given as

(28) $$\begin{array}{c} \left\{\begin{array}{c} {\dot{\hat{W}}}_{i}=-{ϒ}_{w,i}\left[\sigma \left(\chi \right)z_{2i}+k_{w,i}{\hat{W}}_{i}\right],\\ {\dot{\hat{\delta }}}_{i}={ϒ}_{\delta ,i}\left[\frac{z_{2i}{}^{2}}{2w_{i}}-k_{\delta ,i}{\hat{\delta }}_{i}\right], \end{array}\right.\;i=1,\cdots ,n. \end{array}$$

The event-triggering mechanism is designed as

(29) $$\begin{array}{c} \left\{\begin{array}{c} \tau_{i}\left(t\right)=\varpi_{i}\left(t_{k}^{i}\right),\forall t\in \left[t_{k}^{i},t_{k+1}^{i}\right),\\ t_{k+1}^{i}=\inf\left\{t\in \mathbb{R}|\left|e_{\tau i}\right|\geq \Delta_{i}\left|\tau_{i}\left(t\right)\right|+m_{i}\right\}, \end{array}\right. \end{array}$$

where *e*_*τi*_(*t*)=*ϖ*_*i*_(*t*) − *τ*_*i*_(*t*) is the event-triggering errors. The controller update time is defined as *t*_*k*_^*i*^, *k* ∈ *ℝ*^+^, and designed parameters *κ*_*i*_, 0 < Δ_*i*_ < 1*m*_*i*_ > 0, and ${\bar{m}}_{i}>m_{i}/\left(1-\Delta_{i}\right)$ are positive. When time *t*_*k*_^*i*^ ∈ [*t*_*k*_^*i*^, *t*_*k*+1_^*i*^), the controller holds as *ϖ*_*i*_(*t*_*k*_^*i*^) . When triggering condition (29) is triggered, the control signal will be updated and it is marked as *ϖ*_*i*_(*t*_*k*+1_^*i*^). Thus, there exist two continuous time-varying parameters *ρ*_1*i*_(*t*) and *ρ*_2*i*_(*t*) such that *ϖ*_*i*_(*t*)=(1+*ρ*_1*i*_(*t*)Δ_*i*_)*τ*_*i*_(*t*)+*ρ*_2*i*_(*t*)*m*_*i*_ , where |*ρ*_1*i*_(*t*)| ≤ 1 and |*ρ*_2*i*_(*t*)| ≤ 1. Therefore, one gets

(30) $$\begin{array}{c} \tau_{i}\left(t\right)=\frac{\varpi_{i}\left(t\right)-\rho_{2i}\left(t\right)m_{i}}{1+\rho_{1i}\left(t\right)\Delta_{i}}. \end{array}$$

Thus, substituting (30) into (23), the following inequality holds:

(31) $$\begin{array}{c} {\dot{V}}_{3}\leq \sum_{i=1}^{n}-\left(k_{1i}\right)\left[\left(1-q\left(z_{1i}\right)\right)\frac{L_{Li}^{2}}{\pi }\tan\left(\frac{\pi z_{1i}^{2}}{2L_{Li}^{2}}\right)+q\left(z_{1i}\right)\frac{L_{Ui}^{2}}{\pi }\tan\left(\frac{\pi z_{1i}^{2}}{2L_{Ui}^{2}}\right)\right]+\Xi_{\Lambda i}z_{2i}-\frac{e_{fi}^{2}}{t_{d}}-e_{fi}N_{i}\left(\cdot \right)+z_{2i}\left[\frac{\varpi_{i}\left(t\right)-\rho_{2i}\left(t\right)m_{i}}{1+\rho_{1i}\left(t\right)\Delta_{i}}-f_{i}-d_{i}\right]. \end{array}$$

In view of |*ρ*_1*i*_(*t*)| ≤ 1 and |*ρ*_2*i*_(*t*)| ≤ 1, we have

(32) $$\begin{array}{c} \left\{\begin{array}{c} \frac{z_{2i}\varpi_{i}\left(t\right)}{1+\rho_{1i}\left(t\right)\Delta_{i}}\leq \frac{z_{2i}\varpi_{i}\left(t\right)}{1+\Delta_{i}},\\ \frac{-\rho_{2i}\left(t\right)m_{i}}{1+\rho_{1i}\left(t\right)\Delta_{i}}\leq \left|\frac{\rho_{2i}\left(t\right)m_{i}}{1-\Delta_{i}}\right|. \end{array}\right. \end{array}$$

The main results of this paper are given as follows.

**Theorem 1 .**

EL system (1) with uncertainties and Assumptions 1 and 2 are satisfied. Under the actual controller (29) with control law (14), (15), and (25)-(28), the asymmetric constraint tracking control of EL systems can be achieved. All signals in the closed-loop control system are semiglobally uniformly ultimately bounded (SGUUB), and the position error satisfies design objective conditions (2), which can converge to a neighborhood near of origin, and the interexecution intervals *t*_*k*+1_^*i*^ − *t*_*k*_^*i*^ are lower bounded by a nonzero time ${\bar{t}}^{i}>0$, provided that the control parameter satisfies

(33) $$\begin{array}{c} \left\{\begin{array}{c} k_{1i}>0,\\ k_{2i}>0,\\ t_{d}<2, \end{array}\right.\;i=1,\ldots ,n. \end{array}$$

**Proof**

Consider a new Lyapunov function:

(34) $$\begin{array}{c} V=V_{3}+\sum_{i=1}^{n}\frac{1}{2}{\tilde{W}}_{i}^{T}{ϒ}_{w,i}^{-1}{\tilde{W}}_{i}+\frac{1}{2}{ϒ}_{\delta ,i}^{-1}{\tilde{\delta }}_{i}{}^{2}. \end{array}$$

Taking the time derivative of (34) and using (31) and (32), we have

(35) $$\begin{array}{c} \dot{V}\leq \sum_{i=1}^{n}-\left(k_{1i}\right)\left[\left(1-q\left(z_{1i}\right)\right)\frac{L_{Li}^{2}}{\pi }\tan\left(\frac{\pi z_{1i}^{2}}{2L_{Li}^{2}}\right)+q\left(z_{1i}\right)\frac{L_{Ui}^{2}}{\pi }\tan\left(\frac{\pi z_{1i}^{2}}{2L_{Ui}^{2}}\right)\right]+\Xi_{\Lambda i}z_{2i}-\frac{e_{fi}^{2}}{t_{d}}-e_{fi}N_{i}\left(\cdot \right)+z_{2i}\left[\frac{z_{2i}\varpi_{i}\left(t\right)}{1+\left(t\right)\Delta_{i}}+\left|\frac{\rho_{2i}\left(t\right)m_{i}}{1-\Delta_{i}}\right|-f_{i}-d_{i}\right]-{\tilde{W}}_{i}^{T}\left[\sigma \left(\chi \right)z_{2i}+k_{w,i}{\hat{W}}_{i}\right]+{\tilde{\delta }}_{i}\left[\frac{z_{2i}^{2}}{2w_{i}}-k_{\delta ,i}{\hat{\delta }}_{i}\right]. \end{array}$$

The approximate error and disturbances are bounded, and the following equation can be derived:

(36) $$\begin{array}{c} z_{2}^{T}\left(\varepsilon +d+c_{\text{aux}}\right)\leq \sum_{i=1}^{n}z_{2i}\delta_{i}-\frac{{\hat{\delta }}_{i}}{2w_{i}}z_{2i}^{2}\leq \sum_{i=1}^{n}\left(\frac{1}{2w_{i}}z_{2i}^{2}+\frac{w_{i}}{2}\right)\delta_{i}-\frac{{\hat{\delta }}_{i}}{2w_{i}}z_{2i}^{2}\leq \sum_{i=1}^{n}-\frac{{\tilde{\delta }}_{i}}{2w_{i}}z_{2i}^{2}+\frac{w_{i}}{2}\delta_{i}. \end{array}$$

The following inequalities are based on Young's inequality theory, which can be derived as

(37) $$\begin{array}{c} \left\{\begin{array}{c} -k_{w,i}{\tilde{W}}_{i}^{T}{\hat{W}}_{i}\leq -\frac{k_{w,i}}{2}{\left\|{\tilde{W}}_{i}\right\|}_{F}^{2}+\frac{k_{w,i}}{2}{\left\|W_{i}\right\|}_{F}^{2},\\ -k_{\delta ,i}{\tilde{\delta }}_{i}{\hat{\delta }}_{i}\leq -\frac{k_{\delta ,i}}{2}{\tilde{\delta }}_{i}^{2}+\frac{k_{\delta ,i}}{2}\delta_{i}^{2},\\ -e_{fi}N_{i}\left(\cdot \right)\leq \frac{e_{fi}^{2}}{2}+\frac{{\bar{N}}_{i}^{2}}{2}. \end{array}\right. \end{array}$$

According to |*p*| − *p*tanh(*p*/*c*) ≤ 0.2785*c*, for a given variable *p* ∈ *ℝ* and *c* > 0; substituting (26) into (35) gives

(38) $$\begin{array}{c} \dot{V}\leq \sum_{i=1}^{n}-\left(k_{1i}\right)\left[\left(1-q\left(z_{1i}\right)\right)\frac{L_{Li}^{2}}{\pi }\tan\left(\frac{\pi z_{1i}^{2}}{2L_{Li}^{2}}\right)+q\left(z_{1i}\right)\frac{L_{Ui}^{2}}{\pi }\tan\left(\frac{\pi z_{1i}^{2}}{2L_{Ui}^{2}}\right)\right]-k_{2i}z_{2i}^{2}-\left(\frac{1}{t_{d}}-\frac{1}{2}\right)e_{fi}^{2}-\frac{k_{w,i}}{2}{\left\|{\tilde{W}}_{i}\right\|}_{F}^{2}-\frac{k_{\delta ,i}}{2}{\tilde{\delta }}_{i}^{2}+\frac{1}{2}\left({\bar{N}}_{i}^{2}+k_{w,i}{\left\|W_{i}\right\|}_{F}^{2}+k_{\delta ,i}\delta_{i}^{2}+w_{i}\delta_{i}\right)+0.557\kappa_{i}. \end{array}$$

Equation (38) can be expressed as

(39) $$\begin{array}{c} \dot{V}\left(t\right)\leq -\gamma_{1}V\left(t\right)+\gamma_{2}, \end{array}$$

where

(40) $$\begin{array}{c} \left\{\begin{array}{c} \gamma_{1}=\min\left\{k_{1i},\frac{2k_{2i}}{\lambda_{\max}\left(M\right)},\frac{2-t_{d}}{t_{d}},k_{w,i}{ϒ}_{w,i},k_{\delta ,i}{ϒ}_{\delta ,i}\right\},\\ \gamma_{2}=\sum_{i=1}^{n}\frac{1}{2}\left({\bar{N}}_{i}^{2}+k_{w,i}{\left\|W_{i}\right\|}_{F}^{2}+k_{\delta ,i}\delta_{i}^{2}+w_{i}\delta_{i}\right)+0.557\kappa_{i}. \end{array}\right. \end{array}$$

By integration of (39), we have

(41) $$\begin{array}{c} V\left(t\right)\leq V\left(0\right)e^{-\gamma_{1}t}+\frac{\gamma_{2}}{\gamma_{1}}\left(1-e^{-\gamma_{1}t}\right). \end{array}$$

Therefore, the equation is held as follows:

(42) $$\begin{array}{c} 0\leq V_{1}\leq V\leq \frac{\gamma_{2}}{\gamma_{1}}+\left[V\left(0\right)-\frac{\gamma_{2}}{\gamma_{1}}\right]e^{-\gamma_{1}t}. \end{array}$$

The opposite solution of equation (43) is obtained:

(43) $$\begin{array}{c} z_{1i}^{2}\leq \left\{\begin{array}{c} \frac{2L_{Ui}^{2}}{\pi }\tan^{-1}\left\{\frac{\pi }{L_{Ui}^{2}}\left[\frac{\gamma_{2}}{\gamma_{1}}+\left[V\left(0\right)-\frac{\gamma_{2}}{\gamma_{1}}\right]e^{-\gamma_{1}t}\right]\right\}<L_{Ui}^{2},z_{1i}>0,\\ \frac{2L_{Li}^{2}}{\pi }\tan^{-1}\left\{\frac{\pi }{L_{Li}^{2}}\left[\frac{\gamma_{2}}{\gamma_{1}}+\left[V\left(0\right)-\frac{\gamma_{2}}{\gamma_{1}}\right]e^{-\gamma_{1}t}\right]\right\}<L_{Li}^{2},z_{1i}\leq 0. \end{array}\right. \end{array}$$

Therefore, *z*_1_(*t*) remains in the open set *z*_1*i*_ ∈ (−*L*_*Li*_, *L*_*Ui*_), ∀*t* > 0, if the initial state satisfies *z*_1*i*_(0) ∈ (−*L*_*Li*_, *L*_*Ui*_). Then, the tracking error *e*_*p*_ constraints in the time-varying boundary can be implemented.

Motivated by [38, 45], we can prove that there exists time ${\bar{t}}^{i}>0$ such that triggering intervals *t*_*k*+1_^*i*^ − *t*_*k*_^*i*^ is lower bounded by ${\bar{t}}^{i}$. Considering *e*_*τi*_(*t*)=*ϖ*_*i*_(*t*) − *τ*_*i*_(*t*), one has

(44) $$\begin{array}{c} \frac{d}{\text{d}t}\left|e_{\tau i}\right|=\text{sign}\left(e_{\tau i}\right){\dot{e}}_{\tau i}\leq \left|{\dot{\varpi }}_{i}\right|. \end{array}$$

All the signals mentioned above are bounded, and we can get $\left|{\dot{\varpi }}_{i}\right|\leq {\bar{\varpi }}_{i}$, where ${\bar{\varpi }}_{i}$ is a positive parameter. We can obtain that *e*_*τi*_=0 and $\underset{t⟶t_{k+1}}{\lim}e_{\tau i}\left(t\right)=\Delta_{i}\left|\tau_{i}\left(t\right)\right|+m_{i}$ is hold. The lower bound ${\bar{t}}^{i}$ satisfies ${\bar{t}}^{i}\geq \Delta_{i}\left|\tau_{i}\left(t\right)\right|+m_{i}/{\bar{\varpi }}_{i}$. That is, Zeno's behavior is avoided.

This completes the proof.

**Remark 4 .**

The main characteristics of this design are as follows. On the one hand, different from the DSC method [28] without error constraint requirements and the error constant constraint method [22], the error time-varying constraint tracking controller is proposed in this paper, which is stronger in robustness caused by applying the ATBLF technique. On the other hand, different from the fixed structure NN design in [33], the proposed adaptive SSNN can adjust the structure to approximate the nonlinear function with different complexity, and it can reduce the calculation pressure of the system.

## 4. Simulation Results

In this section, the effectiveness of the proposed approach is validated by some simulations. In order to verify the validity of the proposed scheme for EL systems with unknown disturbances and uncertainties, a pendulum and a two-degree-of-freedom robotic manipulator are considered as the experimental plant.

**Example 1 .**

The dynamics model of the pendulum with mass changes is as follows:

(45) $$\begin{array}{c} ml^{2}\ddot{q}+mglq=\tau . \end{array}$$

The model parameter is selected as *m*=2+0.5sin(*t*), *l*=1, and *g*=9.8. The reference trajectories are selected as *q*_*d*_=sin(0.5*t*). The main control parameter is selected as *k*_1_=8, *k*_2_=28, ϒ_*w*_=50, *k*_*w*_=0.1, ϒ_*δ*_=5, *k*_*δ*_=0.1, *w*_1_=0.04, Δ_1_=0.1, *κ*_1_=2.1, *m*_1_=0.1, and ${\bar{m}}_{1}=0.08$. *L*_*L*1_=0.5*e*^−0.8*t*^+0.03, and *L*_*U*_=0.4*e*^−0.8*t*^+0.02. Compared with the traditional PID control, the PID parameter is selected as *P*=300, *I*=20, and *D*=9. The initial state is ${\left[q\left(0\right),\dot{q}\left(0\right)\right]}^{T}={\left[0.3,0\right]}^{T}$.

**Figure 3:**
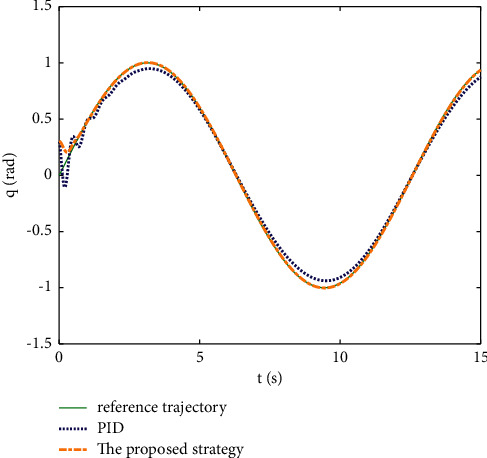
Position tracking of different methods.

**Figure 4:**
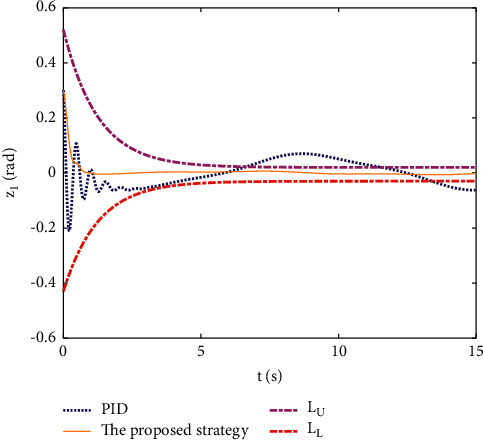
Tracking error of different methods.

**Figure 5:**
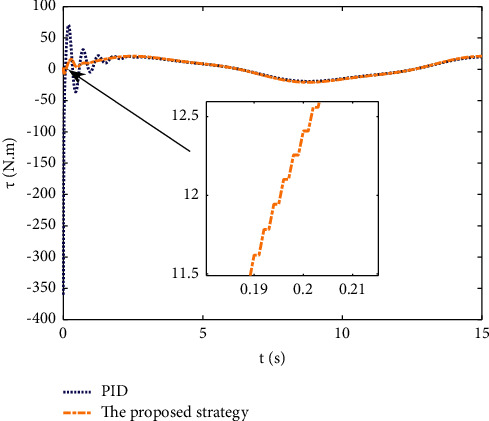
Control input of different methods.

The simulation results of the pendulum tracking are shown in Figures 3–5 .  Figure 3 shows the effect of the two different methods on position tracking. It can be seen that the proposed strategy can stably track the reference trajectory, while the PID control shows some jitter at the beginning, and some steady-state errors exist. The tracking error of two different methods is shown in Figure 4, the error of the proposed strategy converges to near-zero quickly and stably, and the error of PID control exceeds the preset boundary in some time periods. It can be seen that the proposed tracking control strategy is stronger robustness. The control inputs of the two methods are shown in Figure 5. It can be seen that the control input signal of the proposed strategy is updated at intervals, which saves the system communication resources.

**Example 2 .**

The dynamics model of the robotic manipulator can be expressed as (1), where

(46) $$\begin{array}{c} M\left(q\right)=\left[\begin{array}{cc} M_{11} & M_{12}\\ M_{21} & M_{22} \end{array}\right],C\left(q,\dot{q}\right)=\left[\begin{array}{cc} C_{11} & C_{12}\\ C_{21} & C_{22} \end{array}\right],G\left(q\right)=\left[\begin{array}{c} G_{11}\\ G_{21} \end{array}\right], \end{array}$$

(47) $$\begin{array}{c} \left\{\begin{array}{c} M_{11}=\Phi_{1}+\Phi_{2}+2\Phi_{3}\cos q_{2},\\ M_{12}=\Phi_{2}+\Phi_{3}\cos q_{2},\\ M_{21}=M_{12},\\ M_{22}=\Phi_{2},\\ C_{11}=-\Phi_{3}{\dot{q}}_{2}\sin q_{2},\\ C_{12}=-\Phi_{3}\left({\dot{q}}_{1}+{\dot{q}}_{2}\right)\sin q_{2},\\ C_{21}=\Phi_{3}{\dot{q}}_{1}\sin q_{2},\\ C_{22}=0,\\ G_{11}=\Phi_{4}g\cos q_{1}+\Phi_{5}g\cos\left(q_{1}+q_{2}\right),\\ G_{21}=\Phi_{5}g\cos\left(q_{1}+q_{2}\right), \end{array}\right. \end{array}$$

with Φ_1_=*J*_1_+*p*_2_*l*_1_^2^Φ_2_=*J*_2_+0.25*p*_2_*l*_2_^2^, Φ_3_=0.5*p*_2_*l*_1_*l*_2_, Φ_4_=(0.5*p*_1_+*p*_2_)*l*_1_, and Φ_5_=0.5*p*_2_*l*_2_ [4]. The model parameters are selected as *l*_1_=1, *l*_2_=0.95, *p*_1_=0.96, *p*_2_=1.15, *J*_1_=0.21, *J*_2_=0.4, and *g*=9.8.

The reference trajectories are given as follows:

(48) $$\begin{array}{c} \left\{\begin{array}{c} q_{d1}=\sin\left(0.5t\right),\\ q_{d2}=2\cos\left(0.5t\right). \end{array}\right. \end{array}$$

The disturbances are assumed as

(49) $$\begin{array}{c} \left\{\begin{array}{c} d_{1}=1+0.5\sin\left(0.5t\right),\\ d_{2}=0.5+0.5\cos\left(0.5t\right). \end{array}\right. \end{array}$$

Let the tracking error constraint boundary is selected as

(50) $$\begin{array}{c} \left\{\begin{array}{c} L_{L1}=0.5e^{-0.3t}+0.05,L_{U1}=0.4e^{-0.3t}+0.08,\\ L_{L2}=0.3e^{-0.3t}+0.05,L_{U2}=0.5e^{-0.3t}+0.07. \end{array}\right. \end{array}$$

The initial state of the robot is given as *q*(0)=[0.3, 1.7]^*T*^ and $\dot{q}\left(0\right)={\left[0,0\right]}^{T}$. The control parameter is selected:*k*_1_=diag{8,5}, *k*_2_=diag{28,21}, ϒ_*w*,*i*_=50, *k*_*w*,*i*_=0.1, ϒ_*δ*,*i*_=5, *k*_*δ*,*i*_=0.1, *w*_*i*_=0.05, Δ_*i*_=0.15, *κ*_*i*_=2.1, *m*_*i*_=0.1, and ${\bar{m}}_{i}=0.08$, where *i*=1,2. The parameter of self-structuring mechanism is selected: *S*_*s*_=0.75, *S*_*e*_=0.1, *σ*_*e*_=0.3, and *ψ*=0.5; the initial neurons were two.

In order to exhibit the superior performance of the proposed robust tracking control scheme, two existing results are selected for comparison:

1. DSC: this is a general backstepping technique, filter, and adaptive NN, without error constraints, self-structuring methods, and compensators. The main parameters of the DSC controller are given as *k*_1_=diag{8,5}, *k*_2_=diag{28,21}, ϒ_*w*,*i*_=50, and *k*_*w*,*i*_=0.1.
2. Strategy [22]: this is selected as a log-type BLF, filter, and adaptive NN, without self-structuring methods and compensators. The main parameters of the controller are given as *k*_1_=diag{8,5}, *k*_2_=diag{28,21}, ϒ_*w*,*i*_=50, *k*_*w*,*i*_=0.1, *L*_*L*1_=0.55, *L*_*U*1_=0.48, *L*_*L*2_=0.35, and *L*_*U*2_=0.57.

The experimental conditions of the comparative experiment are the same; in the same initial states, there are also inaccurate dynamics models and subject disturbances. The compared method NN uses eleven neurons.

**Figure 6:**
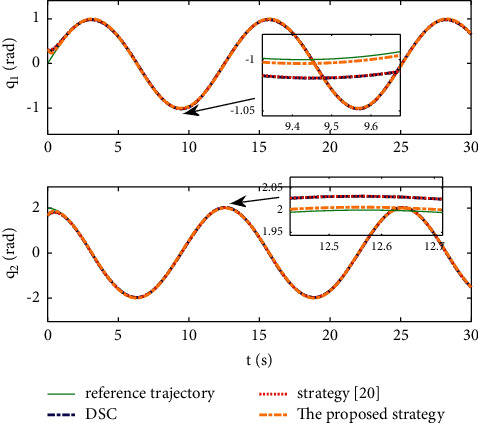
Position tracking with time-varying constraints.

**Figure 7:**
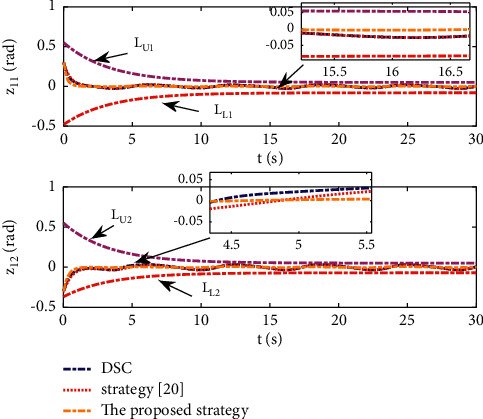
Trajectory tracking error of position.

**Figure 8:**
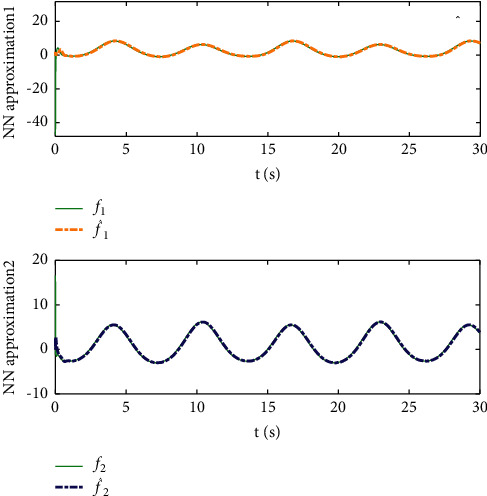
NN approximation uncertainties term.

**Figure 9:**
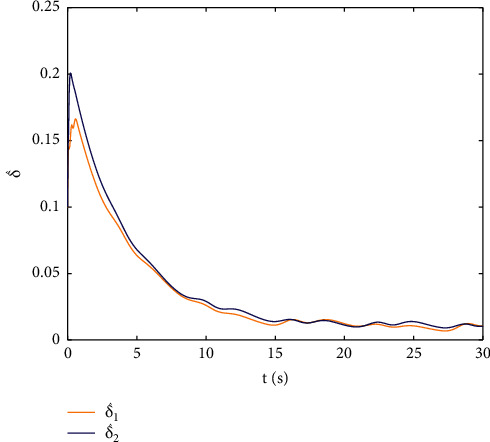
The compensator signed.

**Figure 10:**
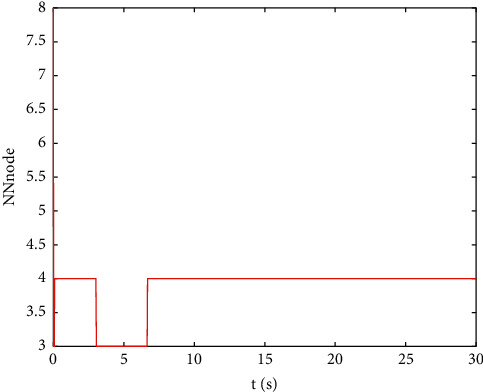
The numbers of neurons.

**Figure 11:**
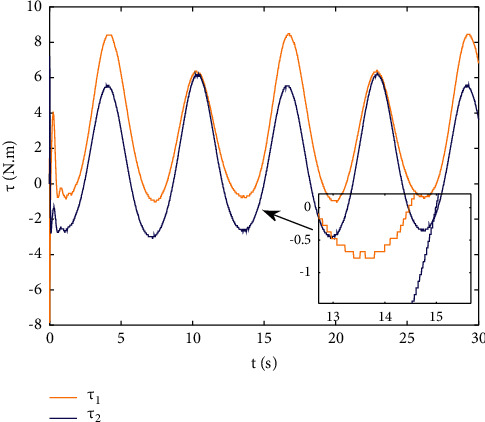
Event-triggered control input.

**Figure 12:**
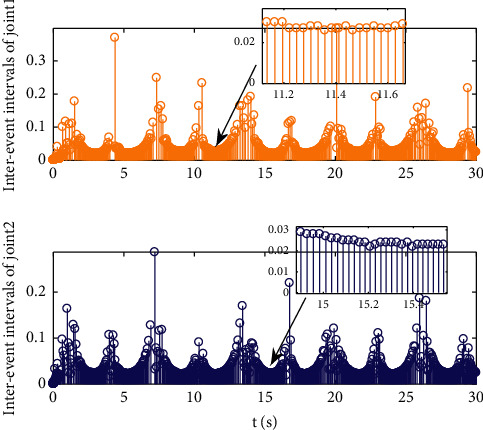
Triggering instants and interevent intervals.

Simulation results of the designed control strategy are shown in Figures 6–12.  Figure 6 shows the movement trajectories of joint 1 and joint 2; we can observe that all the control schemes can track reference trajectories, but it can be seen that the designed control strategy has better tracks accurately. The tracking errors of all control strategies are shown in Figure 7, although there is no violation of the predesign constraint conditions, and the DSC technique and the proposed strategy have a rapid converged rate. However, the proposed method can be tracked accurately and has better robustness when it is subjected to large disturbances. The uncertainties can be accurately estimated by SSNN, as shown in Figure 8.  Figure 9 shows the compensator signal, which proves that the disturbances and the NN estimate errors are bounded.  Figure 10 shows the number of neurons, and the number of neurons after fitting is stable. In the beginning, the initial neuron did not reach the ideal activation value and then the new neurons are split to obtain a better estimate effect. When SSNNs fit the nonlinear part, some redundant neurons are removed to complete an accurate approximation with the optimal number of neurons. Compared with the other two methods, the proposed method not only achieves better tracking performance but also uses fewer neurons on average.  Figure 11 shows the control input, which indicates that it is updated at intervals and is stable and bounded. The trigger times and the trigger intervals of joint 1 and joint2 are shown in Figure 12, and the advantage of cost-saving for the event-triggered controllers is shown.

Consider that the control system is subjected to different disturbances and different dynamics models of the robotic manipulator to verify the robustness of the system and the validity of the SSNN. Another manipulator model is visible in [22]. The three groups' disturbances are set as

(51) $$\begin{array}{c} \left\{\begin{array}{c} d_{11}=0.5\sin\left(t\right),\\ d_{12}=0.5\cos\left(t\right), \end{array}\right.\\ \left\{\begin{array}{c} d_{21}=0.5+2\sin\left(t\right)\cos\left(t\right),\\ d_{22}=0.3+3\sin\left(t\right)\cos\left(t\right), \end{array}\right.\\ \left\{\begin{array}{c} d_{31}=1+0.5\sin\left(0.5t\right)+0.5\left(2\text{rand}\left(1\right)-1\right),\\ d_{32}=0.5+0.5\cos\left(0.5t\right)+0.5\left(2\text{rand}\left(1\right)-1\right). \end{array}\right. \end{array}$$

**Figure 13:**
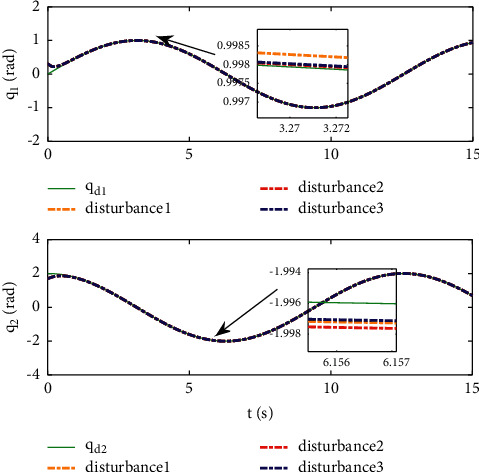
Position tracking performance under different disturbances.

**Figure 14:**
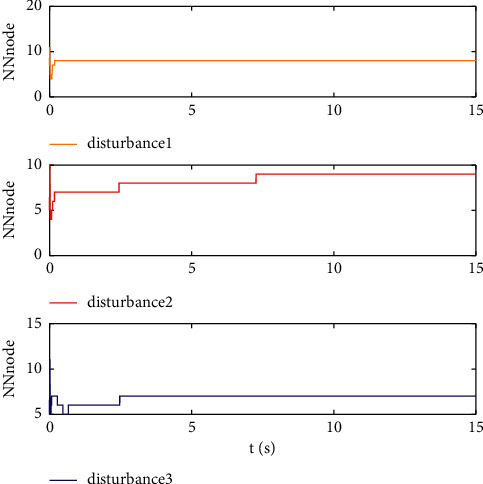
The number of neurons under different disturbances.

The results are shown in Figures 13 and 14 . From Figure 13, one can find that the system still maintains good tracking performance in response to different disturbances.  Figure 14 shows that the structure of SSNN changes according to the complexity of the nonlinear part, but the number of neurons is stable.

**Figure 15:**
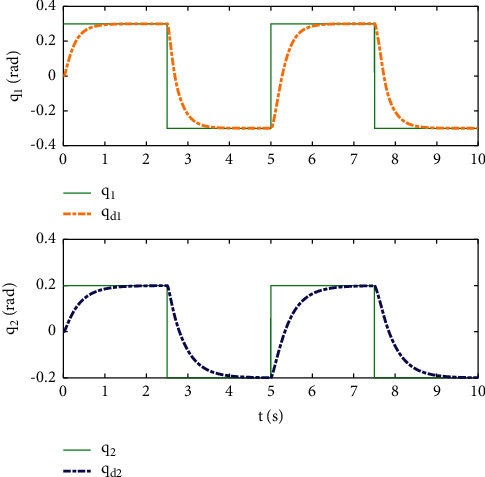
Position tracking of the step experiment.

**Figure 16:**
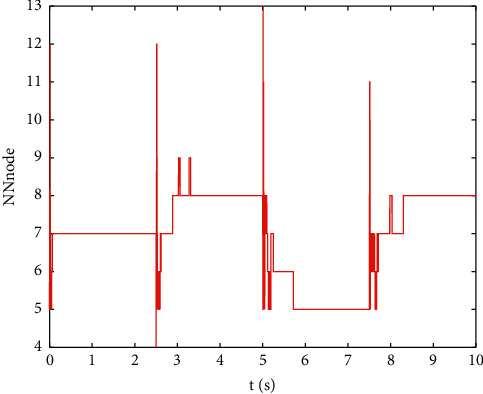
The number of neurons of the step experiment.

Consider a step experiment that switches the tracking signal every 2.5 seconds, and the simulation results are shown in Figures 15 and 16 .  Figure 15 shows the tracking effect of the step signal. It can be seen that the tracking is smooth and the steady-state error is small.  Figure 16 shows that the number of neurons is also stable in the step experiment. These results show that the proposed strategy has good performance.

## 5. Conclusions

This paper studies robust tracking control of EL systems based on BLF and SSNNs with uncertainties. The proposed robust tracking control law consists of the ATBLF method, the SSNNs, the compensator, and the event-triggered methods. The results of stability analysis show that all signals are SGUUB in the closed-loop system. Simulation results show the effectiveness and superiority of the proposed strategy, such as strong robustness and high precision. Further work will include practical experiments and the application of SSNN in the multi-EL system.

## Data Availability

The data used to support the findings of the study can be obtained from the corresponding author upon request.
